# Global research trends and thematic evolution in neoadjuvant therapy and surgery for non-small cell lung cancer: a bibliometric and scientometric analysis

**DOI:** 10.3389/fimmu.2026.1900455

**Published:** 2026-07-29

**Authors:** Xinyu Wu, Wensi Zhao, Xiang Cheng, Ming Li, Huilin Xu, Dedong Cao

**Affiliations:** 1 Department of Oncology, Renmin Hospital of Wuhan University, Wuhan, China; 2 Department of Neurology, Renmin Hospital of Wuhan University, Wuhan, China; 3 Department of Oncology, The Fifth Hospital of Wuhan, Wuhan, Hubei, China

**Keywords:** bibliometrics, immunotherapy, neoadjuvant therapy, non-small cell lung cancer, scientometrics, surgery

## Abstract

**Background:**

Neoadjuvant therapy has become a cornerstone of curative-intent management for resectable non-small cell lung cancer (NSCLC), particularly since immune checkpoint inhibitors and biomarker-guided strategies entered perioperative practice. However, the field’s global knowledge structure, collaboration architecture, and surgery-relevant thematic transitions have not been comprehensively mapped.

**Methods:**

Publications on neoadjuvant/induction/preoperative therapy and surgery for NSCLC (January 1989 to September 2025) were retrieved from the Web of Science Core Collection, PubMed, and Wanfang. Bibliometrix, VOSviewer, and CiteSpace were used to quantify productivity and impact, map collaboration and coupling networks, and identify co-citation clusters and citation/keyword bursts. Latent Dirichlet Allocation (LDA; k=8) was applied to abstracts and keywords to characterize semantic domains and their evolution.

**Results:**

A total of 1,512 publications were included. China and the United States were the dominant contributors and citation hubs. European countries showed higher proportions of multinational collaborations. Core journals formed a dual source landscape bridging thoracic surgery and oncology/immunology. Co-citation clustering showed strong structural validity and highlighted the rise of neoadjuvant chemoimmunotherapy, perioperative safety, and pathological response endpoints. LDA confirmed a shift from induction chemotherapy/chemoradiotherapy toward immunotherapy-centered perioperative paradigms and translational monitoring. Surgery-facing synthesis of representative landmark studies emphasized evolving emphases on complete resection, downstaging, and lung-sparing operative strategies.

**Conclusions:**

From January 1989 to September 2025, neoadjuvant NSCLC research transitioned from cytotoxic induction to immunologically driven, biomarker-informed perioperative paradigms tightly integrated with surgery. This integrated bibliometric–semantic framework provides a reproducible approach to monitor emerging fronts and to translate research signals into clinically interpretable questions for multidisciplinary perioperative decision-making.

## 1 Introduction

Lung cancer remains the leading cause of cancer-related mortality worldwide, accounting for nearly one-fifth of all cancer deaths annually (1). Alongside advances in systemic therapy, perioperative strategies have increasingly complemented surgical resection, shifting practice from single-modality surgery to integrated multimodal management. Neoadjuvant treatment, delivered before definitive resection, is intended to reduce primary tumor burden, increase the likelihood of complete resection, and address occult micrometastatic disease at an early stage.

Before the immunotherapy era, neoadjuvant regimens primarily consisted of cytotoxic chemotherapy with or without radiotherapy, with the objective of improving long-term survival through downstaging and optimized operability. Landmark trials such as EORTC 08941 (2) and INT-0139 (3) supported the feasibility of induction approaches, but their broader adoption was tempered by treatment-related morbidity, variable pathological response, and persistent uncertainty regarding the surgical trade-offs in selected patients.

A major inflection occurred after 2018. Phase II-III studies such as CheckMate 816 (4), NADIM (5), and KEYNOTE-671 (6) demonstrated that immunotherapy-based neoadjuvant regimens could achieve unprecedented rates of pathologic complete response (pCR) and improved event-free survival. Importantly for surgeons, these studies also brought perioperative feasibility, resectability assessment after induction therapy, and the interpretation of residual radiologic findings into sharper clinical focus, reinforcing the need for coordinated multidisciplinary perioperative planning.

Despite rapid progress, the field’s intellectual structure, collaboration networks, and thematic evolution, particularly the way research priorities translate into surgery-facing questions, have not been systematically characterized. Narrative reviews typically synthesize efficacy and safety outcomes but rarely capture macro-level knowledge development, cross-disciplinary connectivity, and emerging fronts over time. Therefore, we used bibliometric and scientometric methods to provide a quantitative and reproducible framework, aiming to map these dynamics and to contextualize research signals relevant to operative decision-making. This study strictly follows the TITAN Guidelines 2025 (**Supplementary Table 1**), including explicit declaration of any artificial intelligence (AI) use and transparent documentation of analytical workflows (7).

## 2 Methods

### 2.1 Data sources and search strategy

A comprehensive literature search was performed on September 30, 2025, using the Web of Science Core Collection (WoSCC), PubMed and Wanfang databases, covering publications from January 1, 1989 to September 30, 2025. To minimize language bias while ensuring analytical consistency, the search strategy was tailored per database (**Supplementary Table 2**). Searches in WoSCC and PubMed were restricted to English. For Wanfang, Chinese-language publications that met the inclusion criteria were then adequately translated into English.

Search terms combined controlled vocabulary and free-text keywords: (“lung cancer” OR “pulmonary neoplasm” OR “bronchogenic carcinoma”) AND (“neoadjuvant therapy” OR “preoperative therapy” OR “induction therapy”) AND (“surgery” OR “resection” OR “operation”). Document types included original articles, reviews, and short communications. Editorials, letters, and conference abstracts were excluded.

### 2.2 Inclusion and exclusion criteria

Inclusion criteria (1): studies focusing on neoadjuvant, induction, or preoperative therapy for lung cancer (2); publications addressing surgical feasibility, pathologic response, or survival outcomes (3); clinical, translational, or scientometric analyses relevant to perioperative management.

Exclusion criteria (1): animal or purely preclinical studies without translational implications (2); meeting abstracts, letters, or non-peer-reviewed content (3); duplicate records or entries with insufficient bibliographic information for analysis.

### 2.3 Data extraction and processing

Two independent investigators screened and extracted bibliographic metadata, including title, authors, institutions, country or region, publication year, keywords, total citations, and journal information. The final dataset was standardized and exported into CSV format for analysis.

To integrate records retrieved from multiple databases, all bibliographic files were imported into CiteSpace (version 6.4.R2) for unified preprocessing. A tiered deduplication strategy was applied prior to all downstream analyses (1): exact DOI matching (primary) (2); title + first author surname + publication year (secondary); and (3) fuzzy title similarity for records lacking DOIs (tertiary). After de-duplication, the cleaned dataset was standardized within CiteSpace and converted into the Web of Science (WoS) plain text format for subsequent bibliometric and downstream analyses. Citation-dependent analyses were performed exclusively on the WoSCC subset, which provides complete and uniformly formatted citation data. Keywords Plus were extracted solely from WoSCC records; for PubMed and Wanfang records, author keywords and database-specific subject headings were used, standardized via a custom synonym thesaurus.

### 2.4 Bibliometric and scientometric analyses

Quantitative bibliometric indicators, such as number of publications (NP), total citations (TC), and H-index, were computed using the bibliometrix (version 5.1) (8). Collaborative networks and co-authorship maps were visualized with VOSviewer (version 1.6.20) (9), while co-citation and burst-detection analyses were conducted in CiteSpace (version 6.4.R2) (10). Modularity (Q) and silhouette (S) metrics were used to evaluate clustering stability and thematic distinctness.

To complement network-based analysis, Latent Dirichlet Allocation (LDA) topic modeling was also implemented in Python to extract latent semantic themes from the titles, abstracts, and author keywords of the included studies. Prior to LDA modeling, a comprehensive text preprocessing was applied to the corpus of titles, abstracts, and author keywords (1): tokenization and removal of punctuation and numerical characters (2); conversion of all text to lowercase (3); removal of common stop words using a custom-built stop word list (4); merging of synonymous keywords and phrases.

The number of topics (k) was determined through empirical optimization of perplexity, evaluating k-values in the range of 4 to 20. The optimal k-value of 8 was selected based on the point where the perplexity curve began to plateau, balancing model fit and complexity (**Supplementary Figure 1**). The model was trained using Gensim with 15 iterations. Automatic asymmetric priors (α = ‘auto’, β = ‘auto’) were applied to adapt topic and word distributions of the data. A fixed random seed (42) ensured reproducibility. Additionally, auto phrase mining was enabled (with a minimum phrase frequency of 3 and a PMI threshold of 8.0) to identify and model meaningful multi-word terms as single semantic units. This integration of structural (CiteSpace) and semantic (LDA) analyses enabled a bidimensional exploration of knowledge evolution, combining the statistical robustness of network metrics with the interpretive depth of thematic patterns.

To translate bibliometric trends into surgical insights, landmark trials were selected for detailed endpoint analysis. There were mainly three selection criteria: high citation impact, thematic representativeness, and Keyword burst strength.

## 3 Results

### 3.1 Search and study selection

**Figure 1:**
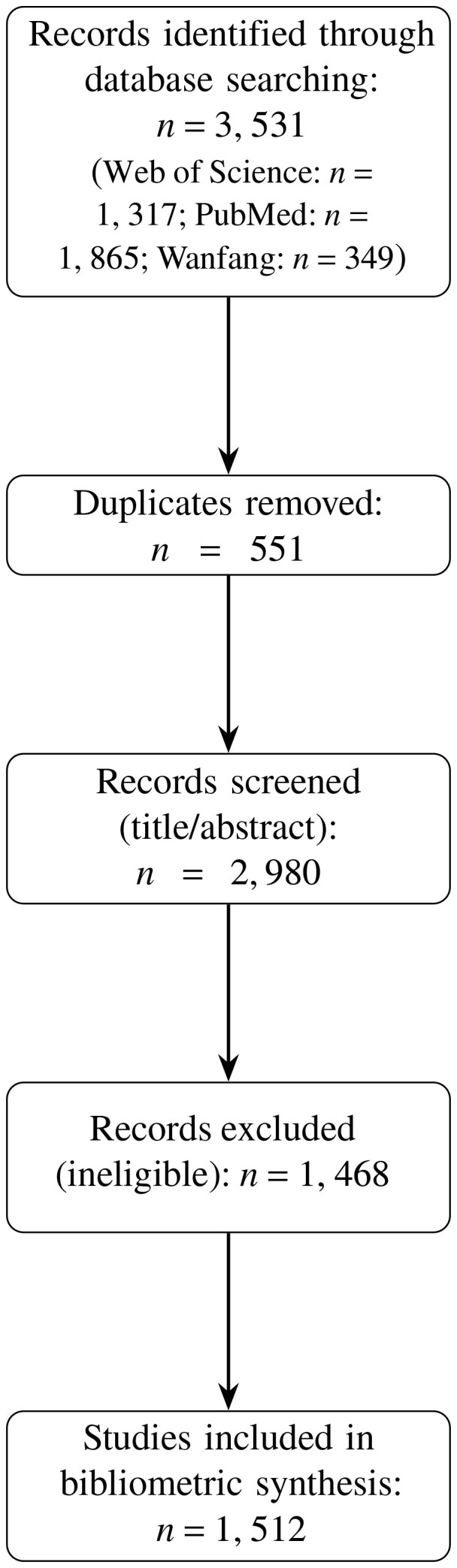
PRISMA-style flow diagram of records identification, screening, and inclusion.

Across three databases, we identified 3,531 records (Web of Science, n=1,317; PubMed, n=1,865; Wanfang, n=349). After removing 551 duplicates, 2,980 unique records underwent title and abstract screening. Of these, 1,468 records were excluded for not meeting the predefined eligibility criteria, leaving 1,512 studies for inclusion in the bibliometric analysis. The study selection process is summarized in **Figure 1**.

Among the included records, we identified 359 unique sources and 25,634 cited references (**Table 1**). The timespan of the data was 1989 to 2025, with a document average age of 8.62 years. In terms of content descriptors, the dataset contained 1,819 Keywords Plus (ID) terms and 1,992 author keywords (DE), providing adequate granularity for downstream co-occurrence and thematic-evolution analyses.

**Table 1:** Main information about the dataset for the bibliometric analysis of neoadjuvant therapy and surgery in lung cancer (1989–2025).

Description	Results
Main information about data	
Timespan	1989:2025
Sources (Journals, Books, etc)	359
Documents	1512
Annual Growth Rate %	14.64
Document Average Age	8.62
Average citations per doc	23.81
References	25634
Document contents	
Keywords Plus (ID)	1819
Author’s Keywords (DE)	1992
Authors	
Authors	7873
Authors of single-authored docs	41
Authors collaboration	
Single-authored docs	47
Co-Authors per Doc	8.2
International co-authorships %	13.49
Document types	
article	1256
proceedings paper*	6
review	250

^a^ ID, indexer-supplied terms (Keywords Plus); DE = author’s keywords.

^b^ International co-authorships (%) = (papers with ≥2 countries/all papers) × 100.

^c^ Articles include original research; reviews include systematic/narrative reviews; proceedings papers denote conference proceedings indexed as such.

^d^ Minor discrepancies may occur due to database indexing updates after the data-lock date.

^*^ Proceedings papers refer to full-length manuscripts published in conference proceedings, not brief meeting abstracts.

### 3.2 Annual publications and citations

**Figure 2:**
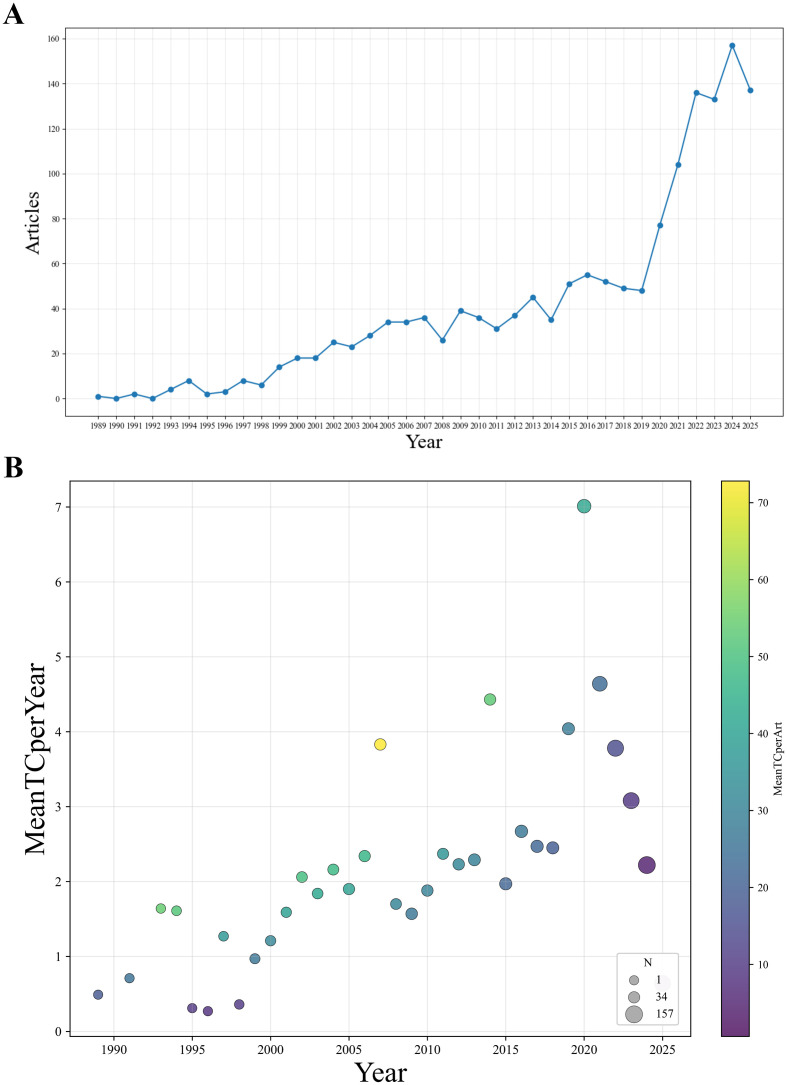
Annual publications and citations (1989–2025). **(A)** Yearly number of publications (NP) on neoadjuvant therapy and surgery in lung cancer, aggregated from Web of Science, PubMed and Wanfang after deduplication. The 2025 data point is incomplete (through September only) and should not be directly compared with full calendar years. **(B)** Bubble plot of citation activity by year. The x-axis is Year; the y-axis is MeanTCperYear (average citations accrued within each calendar year). Bubble size is proportional to the annual number of publications (N), and bubble color encodes MeanTCperArt (average citations per article in that year), jointly summarizing volume, yearly citation intensity, and per-article impact. NP/N, number of publications; MeanTCperYear, mean citations per year; MeanTCperArt, mean citations per article.

From 1989 to 2025, annual output rose from 1 paper in 1989 to 137 papers in 2025 (**Figure 2A**), corresponding to a compound annual growth rate (CAGR) of 14.64% over the entire window. To objectively identify structural changes in the publication trajectory, we applied the Bai–Perron structural break test to the annual publication series. The test identified a statistically significant breakpoint at 2020 (BIC: 339.4 → 269.5; RSS reduction: 88.7%; **Supplementary Figure 5**), rather than a pre-specified year. Segmented linear fits using this data-driven breakpoint revealed that the pre-2020 slope was +2.03 papers per year (CAGR: 15.0%), which increased sharply to +13.03 papers per year (CAGR: 12.2%) after 2020, representing a slope increment of +11.00 papers per year.

Year-specific averages indicated that the mean citations per article (MeanTCperArt) across all years was 30.27 (**Figure 2B**). As expected from citation-window effects, the pre-2020 (1989–2020) period had a higher mean (33.58) than the post-2020 (2021–2025) period (10.53). Year-level citation activity (MeanTCperYear) similarly increased alongside publication growth; high-citation years concentrated from 2020 onward, overlapping with the dissemination of pivotal randomized trials in neoadjuvant immunotherapy.

### 3.3 Sources and publication outlets

**Figure 3:**
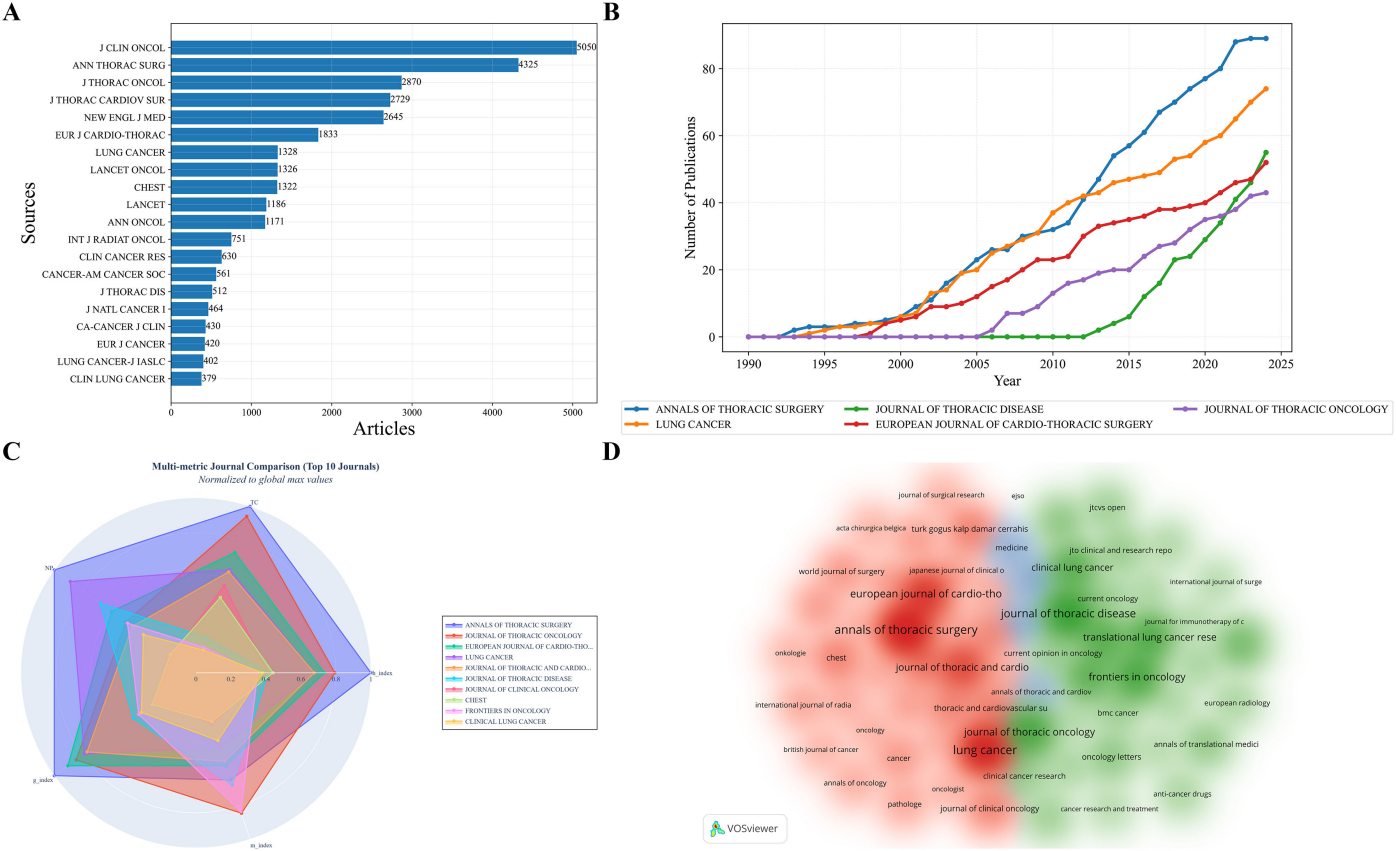
Sources landscape and metrics. **(A)** Most cited sources: outlets ranked by total citations. **(B)** Five most productive journals (NP) with article-level impact indicators. **(C)** Multi-metric radar comparison for the Top 10 journals, showing normalized (0–1) scores on h_index, TC, NP, g_index, m_index; larger radial extent indicates stronger performance on that metric. **(D)** VOSviewer bibliographic coupling density map of sources: color intensity = local link density; twin density peaks at surgery and oncology/immune cores. NP, number of publications; TC, total citations; h_index, Hirsch index.

A total of 359 distinct sources contributed to the analyzed dataset (**Figure 3A**). The distribution of output was notably concentrated within a small cluster of chest-surgery and thoracic oncology journals. The five most productive journals, ranked by their total number of publications (NP), are presented in **Figure 3B**. And the Top 20 core sources and their citation influence are listed in **Table 2**. While *Annals of Thoracic Surgery* and *Lung Cancer* were the leading contributors in terms of volume, *Journal of Thoracic Oncology* exhibited the highest citation impact per article, despite its smaller publication count. Similarly, the *European Journal of Cardio-Thoracic Surgery* demonstrated strong article-level impact, affirming the critical role of these specialized venues in disseminating highly impactful research (**Figure 3C**). The application of Bradford’s Law of Scattering also confirmed a classic core–periphery distribution pattern for the literature (**Supplementary Figure 2**).

**Table 2:** Top 20 core sources and their citation influence.

Source	NP	TC	Average citations
ANNALS OF THORACIC SURGERY	89	3155	35.45
LUNG CANCER	79	1955	24.75
JOURNAL OF THORACIC DISEASE	60	667	11.12
EUROPEAN JOURNAL OF CARDIO-THORACIC SURGERY	53	2281	43.04
JOURNAL OF THORACIC ONCOLOGY	44	2967	67.43
FRONTIERS IN ONCOLOGY	43	483	11.23
JOURNAL OF THORACIC AND CARDIOVASCULAR SURGERY	40	1914	47.85
TRANSLATIONAL LUNG CANCER RESEARCH	38	361	9.5
THORACIC CANCER	34	164	4.82
CLINICAL LUNG CANCER	33	429	13
CANCERS	30	264	8.8
FRONTIERS IN IMMUNOLOGY	21	181	8.62
INTERACTIVE CARDIOVASCULAR AND THORACIC SURGERY	19	270	14.21
TURK GOGUS KALP DAMAR CERRAHISI DERGISI-TURKISH JOURNAL OF THORACIC AND CARDIOVASCULAR SURGERY	16	20	1.25
CHEST	16	1428	89.25
THORACIC AND CARDIOVASCULAR SURGEON	15	71	4.73
JOURNAL OF CLINICAL ONCOLOGY	15	1657	110.47
ANTICANCER RESEARCH	14	106	7.57
ANNALS OF SURGICAL ONCOLOGY	13	427	32.85
ONCOLOGY LETTERS	13	44	3.38

NP, number of publications; TC, total citations.

#### 3.3.1 Bibliographic coupling of sources (VOSviewer)

The density view (**Figure 3D**) reveals two macro-clusters: a thoracic-surgery–centric group (e.g., *Annals of Thoracic Surgery*) and an oncology or immune-centric group (e.g., *Lung Cancer*, *Journal of Thoracic Oncology*). Several bridge nodes (e.g., *Journal of Thoracic and Cardiovascular Surgery*) connect these clusters, indicating cross-domain citation flows.

### 3.4 Countries and regions

**Figure 4:**
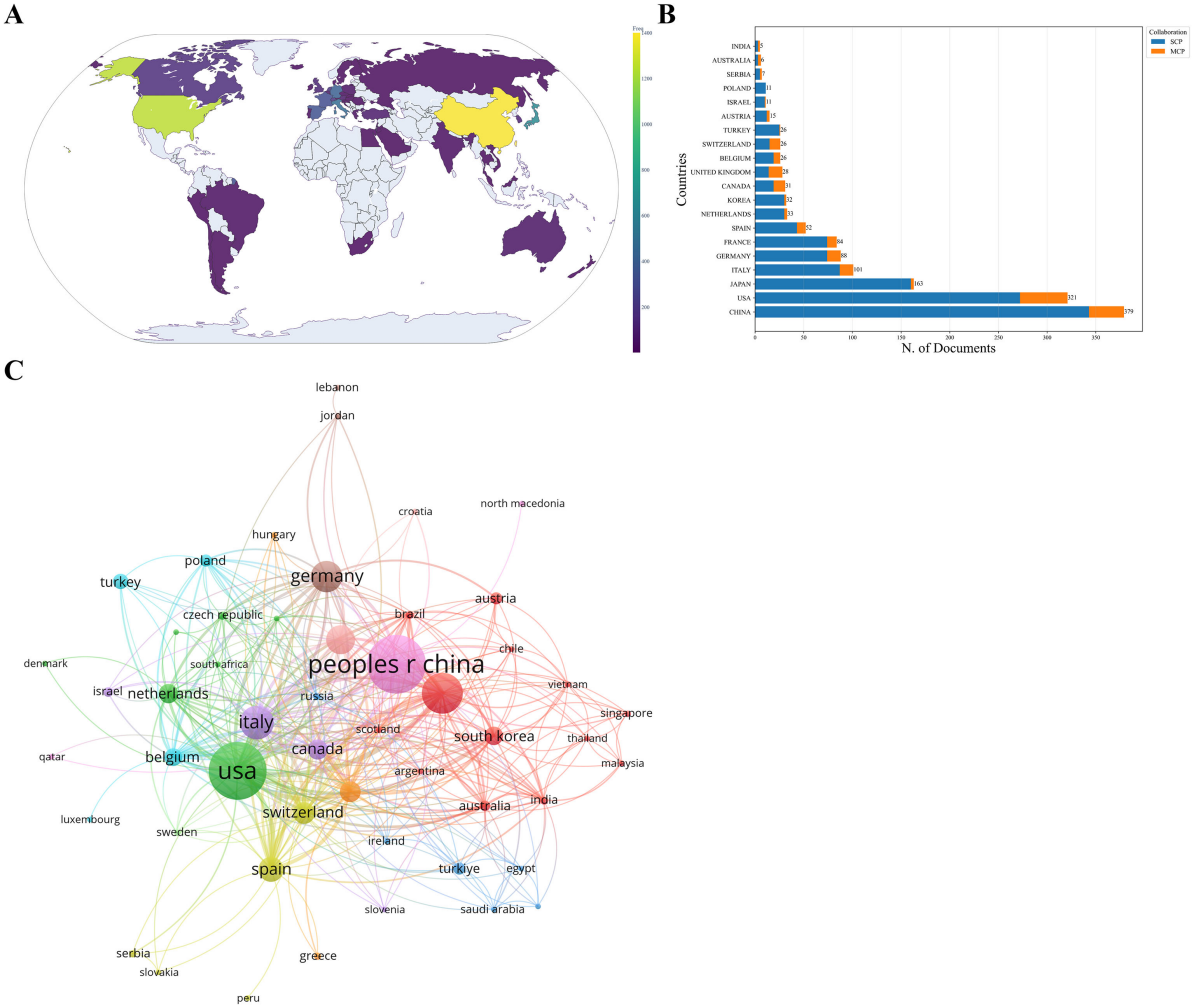
Country-level scientific landscape and collaboration. **(A)** Global distribution of publication output by country (heatmap). **(B)** Stacked bar chart of Single-Country Publications (SCP) vs Multiple-Country Publications (MCP) by corresponding-author country, highlighting national vs international collaboration proportions. **(C)** Co-authorship map displaying regional clusters and major cooperative ties. NP, number of publications; TC, total citations; TLS, total link strength; MCP, multiple-country publications; SCP, single-country publications.

A total of 56 countries contributed to publications in the field (**Figure 4A**). The Top 10 productive countries and their citation influence are listed in **Table 3**. While China led the world in total publication count, its average citation impact was lower than that of the United States and major European countries. The analysis of most relevant countries by corresponding author (**Figure 4B**) suggested that China showed the highest absolute number of single-country publications (SCP) and relatively fewer multiple-country publications (MCP). In contrast, the United States, Italy, Germany, France, Switzerland, and England displayed a higher proportion of MCP. European countries demonstrated the strongest integration into multinational collaborations.

**Table 3:** Top 10 most productive countries and regions.

Country	NP	TC	Average article citations
CHINA	379	4873	12.9
USA	321	11819	36.8
JAPAN	163	3463	21.2
ITALY	101	1636	16.2
GERMANY	88	2253	25.6
FRANCE	84	1962	23.4
SPAIN	52	1433	27.6
NETHERLANDS	33	839	25.4
KOREA	32	344	10.8
CANADA	31	804	25.9

NP, number of publications; TC, total citations.

#### 3.4.1 Co-authorship network (VOSviewer-collaboration analysis)

The co-authorship map (**Figure 4C**) revealed three macro-level cooperation zones. The trans-Pacific cluster, dominated by China, the United States, and Japan, reflecting frequent collaborations on large clinical trials and translational studies. The European cluster, led by Italy, Germany, France, Spain, Switzerland, and England, with high internal connectivity and balanced output-impact ratios. The emerging cooperation cluster, including South Korea, Australia, Canada, and India, linking regional initiatives to the two dominant blocs.

### 3.5 Authors and teams

#### 3.5.1 Productivity and influence

**Figure 5:**
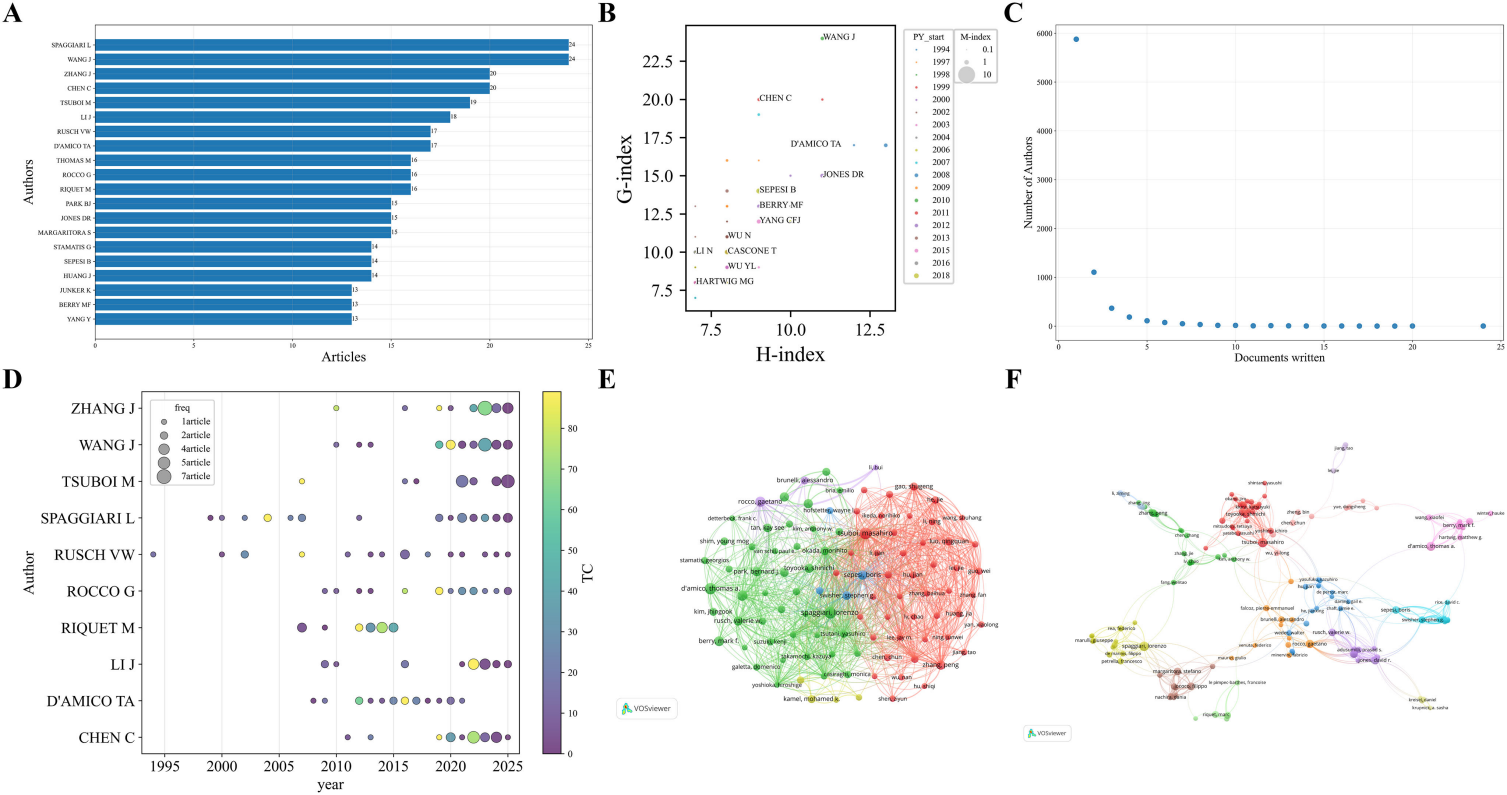
Author and team ecosystem in neoadjuvant lung cancer research. **(A)** Most productive authors (Top 20 by NP). **(B)** Most influential authors (Top 20 by LC/GC, h/g/m indices). **(C)** Lotka’s law fit for author productivity distribution. **(D)** Temporal evolution of author productivity over time. **(E)** Bibliographic coupling-authors network (VOSviewer). **(F)** Co-authorship network among authors. NP, number of publications; TC, total citations; LC, local citations; GC, global citations; TLS, total link strength; h/g/m, Hirsch/g/m-indices; Q, modularity coefficient; MCP, multiple-country publications; SCP, single-country publications; R², coefficient of determination (Lotka fit).

A total of 7,873 authors contributed to publications in the analyzed corpus. The Top 20 most productive authors (NP ≥ 5) are visualized in **Figure 5A**. In terms of citation impact, several researchers, such as Thomas A. D’Amico, Valerie W. Rusch, and David R. Jones, achieved higher citation counts, H-index, G-index, and M-index, respectively. Asian investigators such as Yi-Long Wu, demonstrated rapid growth and strong total link strength in bibliographic coupling and co-authorship networks. The author impact indices are presented in **Figure 5B**.

#### 3.5.2 Lotka’s law and temporal productivity

The Lotka law fitting (**Figure 5C**) demonstrated a good fit to a power-law model (R² = 0.94), suggesting a productivity distribution consistent with that observed in mature biomedical research domains. The author production over time analysis (**Figure 5D**) demonstrated a marked expansion of active authors after 2018.

#### 3.5.3 Author-level coupling networks

The coupling network (**Figure 5E**) revealed two interlinked cores: a Western surgical research hub centered on Rusch V.W., Spaggiari L., D’Amico T.A., and Swisher S.G., and an East-Asian translational cluster anchored by Wu Y.L., Zhong W.Z., and Gao S. Cross-regional citation ties between these hubs demonstrated a well-connected international knowledge exchange structure.

#### 3.5.4 Co-authorship structure and collaboration density

The co-authorship network (**Figure 5F**) contained 214 nodes in the largest connected component, with average degree = 3.6, network density = 0.017, and modularity Q = 0.71. Distinct collaboration clusters corresponded to thematic domains. Cluster 1: surgical innovation + perioperative immunotherapy; Cluster 2: targeted and molecular therapy; and Cluster 3: translational trials and multicenter coordination. According to the most relevant authors dataset (**Supplementary Figure 3**), corresponding authors were predominantly affiliated with China, Japan, and the United States.

### 3.6 Documents

#### 3.6.1 Local and global citation performance

**Figure 6:**
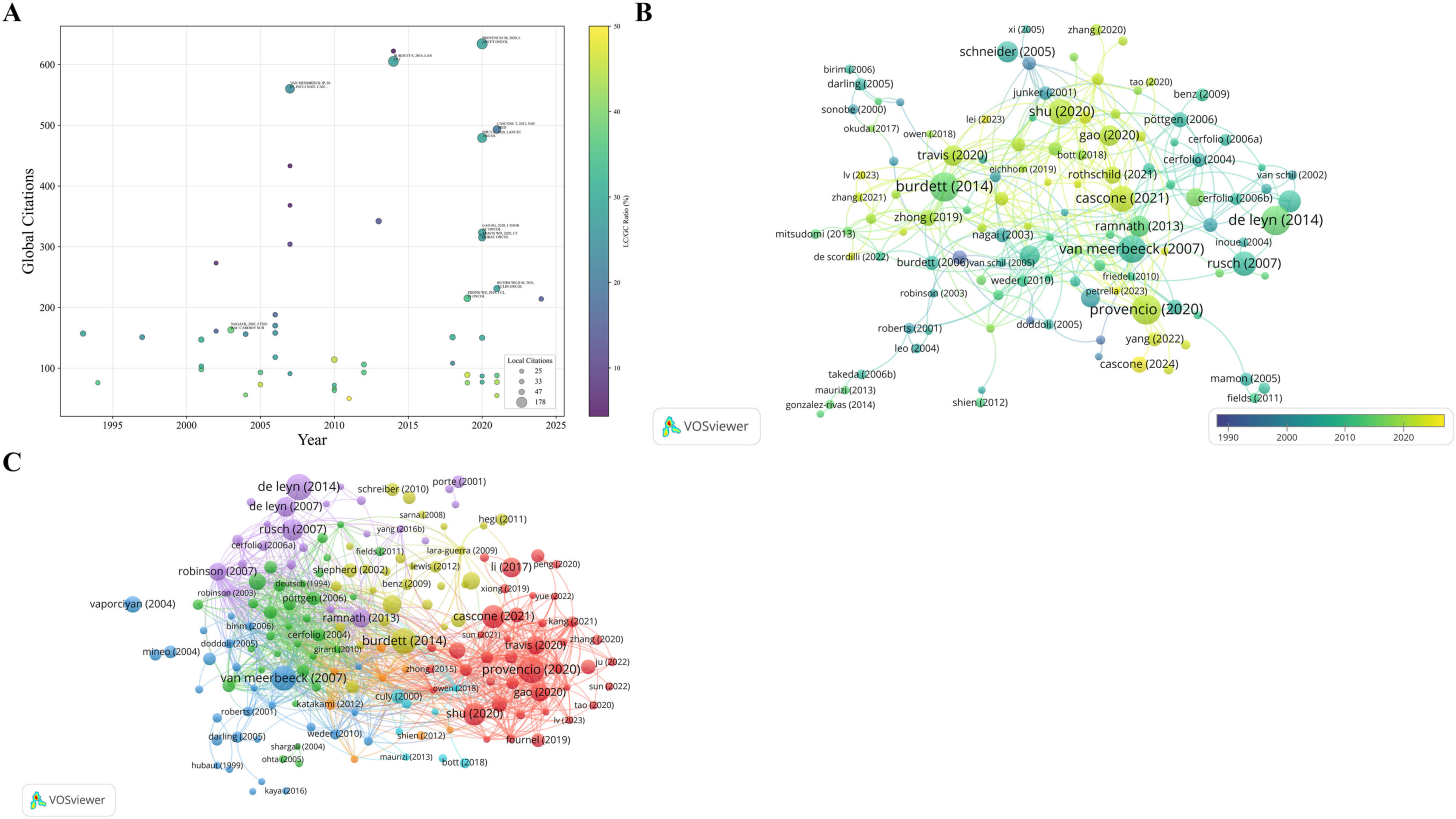
Document-level impact and thematic structure. **(A)** The most locally and globally cited documents ranked by citations. **(B)** Citation network of documents (VOSviewer). **(C)** Bibliographic-coupling map of documents highlighting post-2018 phase II–III trial interconnectivity. LC, local citations; GC, global citations; TLS, total link strength; pCR, pathologic complete response; NP, number of publications.

The most locally cited and globally cited papers are visualized in **Figure 6A**. The locally cited corpus highlighted classical surgical studies such as Rusch (1994) (11), Junker (1997, 2001) (12, 13), Van Meerbeeck (2007) (2), and De Leyn (2007) (14), which collectively shaped the historical foundation of neoadjuvant thoracic surgery. The globally cited list was dominated by recent immuno-oncology phase II-III trials, including Provencio (2023, Nivolumab + Chemotherapy, NEJM) (15), Cascone (2021, Nivolumab + Ipilimumab) (16), and Zhong (2019, Targeted Epidermal Growth Factor Receptor Tyrosine Kinase Inhibitor (EGFR-TKI) Neoadjuvant Study) (17). These trials accumulated GC > 400 citations within 4 years, reflecting their paradigm-shifting influence.

#### 3.6.2 Citation and coupling relationships among documents

The VOSviewer citation network (**Figure 6B**) showed two dominant citation clusters: a surgical-perioperative cluster, forming the traditional evidence base of surgical resectability and adjuvant sequencing; a molecular-immunotherapy cluster, forming the contemporary translational evidence base since 2018. Cross-links between these clusters highlight a clear intellectual transition from classical surgical optimization to integrated immuno-oncology paradigms. The bibliographic coupling network (**Figure 6C**) revealed that Provencio (2020) (5), Cascone (2021) (16), and Shu (2020) (18) exhibited the highest total link strength (TLS > 200), followed by Van Meerbeeck (2007) (2) and De Leyn (2007) (14) as enduring reference anchors.

### 3.7 Reference coupling and co-citation analysis

#### 3.7.1 Co-citation clustering (CiteSpace)

**Figure 7:**
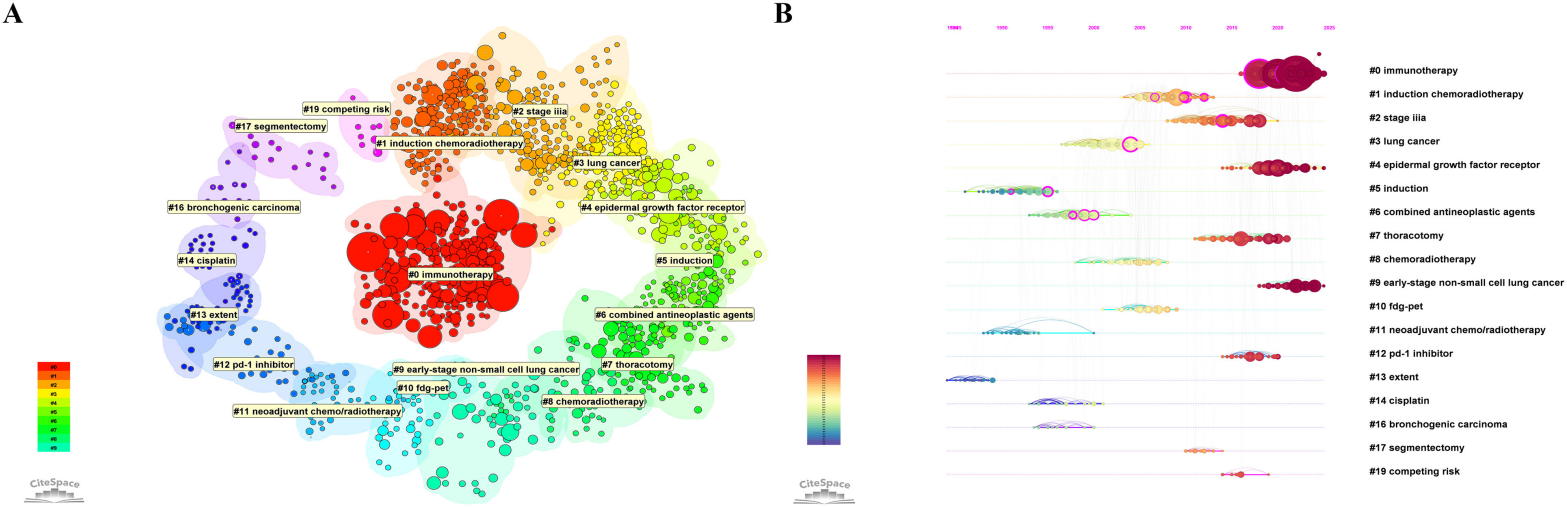
Reference coupling and co-citation network analysis. **(A)** CiteSpace clustering of cited references (Q = 0.712, S = 0.936). **(B)** Timeline view illustrating thematic evolution from chemoradiotherapy (2010 to 2017) to immunotherapy dominance (2018 to 2025).

The co-citation network of references generated by CiteSpace comprised 21 clusters, exhibiting robust modularity (Q = 0.712) and high mean silhouette (S = 0.936), indicating significant structural validity and thematic consistency (**Figure 7A**).

Major clusters reflected the evolving therapeutic paradigms in neoadjuvant lung cancer management. Cluster #0 (“induction chemo-immunotherapy”) represented the largest and most cohesive community (n = 220, S = 0.888), encompassing milestone trials such as Forde PM et al., NEJM 2022 (4), Provencio M et al., Lancet Oncol 2020 (5), and Wakelee H et al., NEJM 2023 (19), which established immune-chemotherapy combinations as the new perioperative standard. Cluster #1 (“10-year survival/historical multimodality”) (n = 149, S = 0.848) traced earlier chemoradiotherapy-based induction strategies, represented by Albain KS et al., Lancet 2009 (3) and Pignon JP et al., J Clin Oncol 2008 (20). Cluster #2 (“stage IIIA/multidisciplinary tumor board”) (n = 136, S = 0.919) encompassed works addressing locally advanced resectable disease and the evolving role of adjuvant versus neoadjuvant sequences [e.g., Antonia SJ et al., NEJM 2018 (21); Postmus PE et al., Ann Oncol 2017 (22)]. Cluster #4 (“EGFR-TKI induction”) (n = 77, S = 0.938) reflected the targeted-therapy era, dominated by epidermal growth factor receptor–focused adjuvant strategies [e.g., Felip E et al., Lancet 2021 (23)]. Cluster #7 (“pCR”) (pCR/VATS, video-assisted thoracoscopic surgery) (n = 65, S = 0.952) linked surgical innovation with immune-induced tumor regression and the adoption of minimally invasive resections.

The timeline view (**Figure 7B**) revealed two distinct developmental phases (1): 2010–2017, dominated by conventional chemotherapy or chemoradiotherapy regimens; and (2) 2018–2025, characterized by a sharp thematic shift toward immuno-chemotherapy and perioperative immune modulation, coinciding with pivotal phase III trials [CheckMate 816 (4), AEGEAN (24), KEYNOTE-671 (6)].

#### 3.7.2 Citation burst detection

Citation burst analysis identified successive waves of landmark publications shaping the domain’s intellectual structure. In the most recent five years (2018 to 2025), the strongest citation bursts were observed (**Table 4**).

**Table 4:** Top 5 Citation Bursts (2018–2025).

Reference	Year	Strength	Burst period	Key contribution
Forde PM, N Engl J Med 2018 (33)	2018	45.55	2019–2023	Introduced neoadjuvant nivolumab + chemotherapy (CheckMate 816 trial)
Antonia SJ, N Engl J Med 2018 (21)	2018	15.10	2019–2023	Established consolidation immunotherapy post-CRT paradigm
Provencio M, Lancet Oncol 2020 (5)	2020	17.80 (est.)	2021–2024	NADIM I/II: confirmed feasibility of preoperative ICI + CT
Zhong WZ, J Clin Oncol 2019 (17)	2019	11.94	2020–2025	EGFR-TKI neoadjuvant feasibility (EMERGING-CTONG 1103)
Heymach JV, N Engl J Med 2023 (24)	2023	13.20 (est.)	2023–2025	AEGEAN: durvalumab perioperative standardization

CRT, chemoradiotherapy; ICI, immune checkpoint inhibitor; CT, chemotherapy; EGFR-TKI, Epidermal Growth Factor Receptor Tyrosine Kinase Inhibitor.

### 3.8 Keywords

#### 3.8.1 Keyword co-occurrence network

The co-occurrence network contained 1,423 unique keywords, with 482 nodes exceeding the minimum threshold. The selection of the threshold was informed by a sensitivity analysis (**Supplementary Figure 4**). The value of 10 was identified as the point at which modularity (Q) reached a plateau and the number of isolated nodes stabilized, ensuring network sparsity and interpretability while maintaining structural integrity.

**Figure 8:**
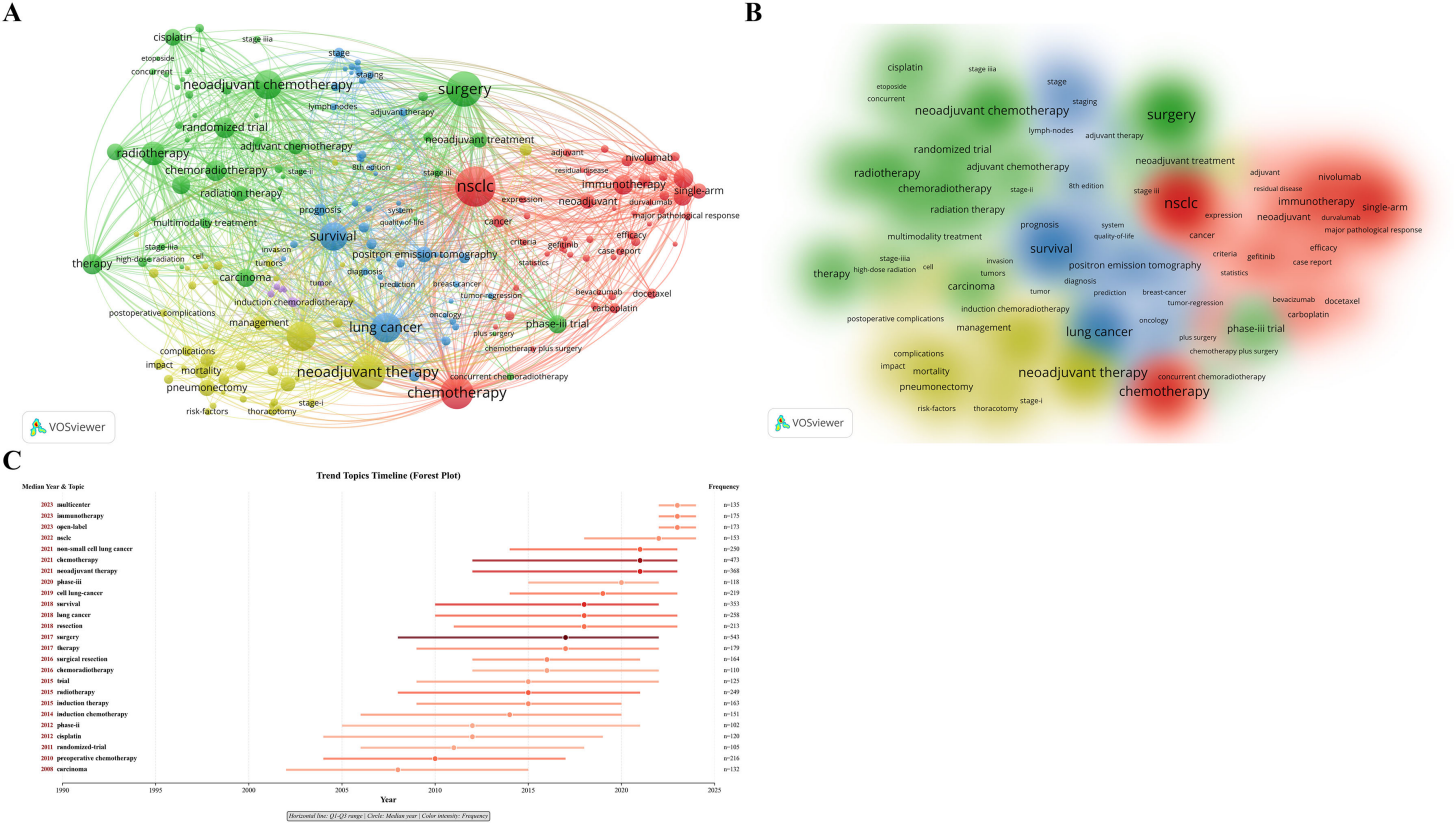
Keyword co-occurrence and thematic evolution in neoadjuvant lung cancer research. **(A)** Keyword co-occurrence network (VOSviewer) **(B)** Density map (VOSviewer) **(C)** Trend-topic timeline (bibliometrix).

The top 50 keywords are presented in **Figure 8A**. The density map (**Figure 8B**) revealed four major conceptual domains: therapeutic strategies (chemotherapy + immunotherapy); surgical outcomes and pathological evaluation (pCR, major pathologic response (MPR), complete resection (R0)); biomarker-driven approaches (circulating tumor DNA (ctDNA), Programmed Death-Ligand 1 (PD-L1), tumor mutational burden (TMB)); and perioperative safety and management (morbidity, complications).

#### 3.8.2 Keyword burst detection

Keyword burst detection identified successive waves of emerging interest. During 1990 to 2010s, classic terms such as “carcinoma”, “cisplatin”, and “randomized trial” dominated early bursts. After 2018, rapid shifts occurred toward clinical trial design and immunotherapy-related concepts (**Figure 8C**).

### 3.9 Surgical endpoints mapping

**Tables 5**, **6** summarize representative neoadjuvant studies in resectable non-small cell lung cancer (NSCLC), with a focus on surgery-facing endpoints and how operative patterns differ across cohorts.

**Table 5:** Surgical endpoints from neoadjuvant studies in resectable non-small cell lung cancer (NSCLC).

Study	Study design	Neoadjuvant regimen	Sample size (ITT)	Surgical resection	R0 rate	Perioperative complications/mortality	Key surgical takeaway
Provencio et al., NADIM (2020, Lancet Oncol) (5)	Phase II, open-label, single-arm, multicenter	Nivolumab + carboplatin/paclitaxel (3 cycles), surgery, adjuvant nivolumab	46	41 underwent resection	41 radical (R0) resection	Surgical complications: 12/41 (29%); mortality NR	High feasibility and R0 rate after chemo-IO.
Cascone et al., NEOSTAR (2021, Nat Med) (16)	Phase II, randomized (Nivolumab vs Nivolumab+Ipilimumab); perioperative comparative analysis	Nivolumab ± ipilimumab (immune-only arms) then surgery	44	37 completed surgery	37 radical (R0) resection	Perioperative outcomes acceptable and manageable; mortality NR	Neoadjuvant ICI did not preclude surgery; safety acceptable.
Shu et al. (2020, Lancet Oncol) (18)	Phase II, single-arm	Atezolizumab + carboplatin + nab-paclitaxel → surgical resection	30	29 resection completed except 1 (progression)	26 radical (R0) resection	No treatment-related surgical delay; 30-day mortality: ≈3% (1/29)	Chemo-IO feasible; high OR access, high R0, meaningful downstaging.
EORTC 08941 surgery arm (Van Schil/Van Meerbeeck et al., ERJ 2005) (25)	Phase III (responders to induction cisplatin-based chemo randomized to surgery vs RT); surgical arm analysis	Induction platinum chemotherapy → surgery	167 randomized to surgery (≈149 evaluable for resection metrics)	149 completed surgery	74 radical (R0) resection	re-operation ≈8.1%; 30-day mortality ≈4.0% (6/149); 90-day mortality ≈8.7% (13/149);	Selective surgery feasible with non-trivial risk; careful selection needed.
Xiong et al. (2019, The Oncologist) (38)	Prospective, single-arm, phase II	Erlotinib 150 mg daily for 56 days, then surgery; adjuvant erlotinib	19	14 underwent surgery	13 radical (R0) resection	NR for 30/90-day mortality; AEs mainly rash; tolerated	Neoadjuvant erlotinib feasible; improved radical resection rate in EGFR+ IIIA-N2.
EMERGING-CTONG 1103 (2019 JCO primary; 2023 STTT final OS)	Randomized, open-label, phase II (Erlotinib vs Gemcitabine/Cisplatin)	Erlotinib (42 days) vs GC (2 cycles), then surgery; adjuvant per protocol	72 (E 37/GC 35)	55 (E 31/GC 24) underwent surgery	49 (E 27/GC 22) radical (R0) resection	Grade ≥3 AEs lower with erlotinib perioperatively; serious AEs rare	Targeted neoadjuvant clinically feasible; perioperative toxicity favorable vs GC.
INT-0139/RTOG-9309 (2009, Lancet; JTCVS periop analysis)	Randomized phase III (Induction chemoradiation → surgery vs definitive chemoradiation)	Cisplatin/etoposide + CRT → surgery (lobectomy/pneumonectomy)	202 included in the analysis	164 underwent surgery	144 radical (R0) resection	High mortality with pneumonectomy post−CRT	CRT→surgery feasible; lobectomy preferred; caution with pneumonectomy.
NeoADAURA (2025, JCO/ASCO Post)	Phase III, randomized (Osimertinib+Chemo vs Osimertinib vs Placebo+Chemo)	Osimertinib ± platinum-based chemotherapy vs placebo + chemotherapy	358 total (121/117/120)	331 completed surgery: 111 (Osi+CT), 113 (Osi), 107 (Pbo+CT)	307 radical (R0) resection: 101 (Osi+CT), 107 (Osi), 99 (Pbo+CT)	Most AEs were low grade; 30-day mortality = 0;	High surgery and R0 rates across arms; perioperative feasibility favorable.
Durvalumab ± SBRT window (2021, Lancet Oncol)	Phase II, randomized window-of-opportunity (Durvalumab vs Durvalumab+SBRT) before surgery	Single-cycle durvalumab ± short-course SBRT → surgery	60 total	52 completed surgery: 26 (durvalumab), 26 (durvalumab + SBRT)	NR	Grade 3 surgical complications occurred in 20 patients (10 in each group)	Adding SBRT did not preclude surgery; perioperative feasibility acceptable.
Concurrent CRT + Pembrolizumab → surgery (2022, JTO Clin Res Rep)	Phase I, single-arm	Cisplatin/etoposide + 45 Gy/25 fx thoracic RT + pembrolizumab → surgery	9	6 completed surgery	6 radical (R0) resection	2 cases of perioperative complications occurred, both fully recovering	Triple−modality feasible; prospective safety acceptable in early study.
ALNEO (2025 ASCO final; JCO abstract)	Phase II, single-arm, multicenter (ALK+ stage III, resectable)	Alectinib (neoadjuvant) → surgery → adjuvant alectinib	33	28 completed surgery	24 radical (R0) resection	NR	ALK−TKI perioperative approach feasible with promising MPR; surgical feasibility high.
Camrelizumab + Chemo vs Chemo (2023, JAMA Oncol)	Randomized phase II (resectable IIIA/selected IIIB)	Camrelizumab + nab−paclitaxel + platinum vs chemotherapy alone → surgery	88	82 completed surgery: 40 (IO+Chemo), 42 (Chemo)	73 radical (R0) resection: 37 (IO+Chemo), 36 (Chemo)	Perioperative death 1/40 (2.5%) in camrelizumab+chemo; complications listed (e.g., bleeding, infection)	Adding PD−1 maintained surgical feasibility; perioperative events acceptable; pCR improved.
CheckMate 816 (2022, N Engl J Med)	open-label, phase III trial (resectable stage IB to IIIA NSCLC)	Nivolumab plus platinum vs. Platinum-based chemotherapy alone → surgery	358	284 completed surgery: 149 (IO+Chemo), 135 (Chemo)	229 radical (R0) resection: 124 (IO+ Chemo), 105 (Chemo)	NR	Nivo + Chemo vs Chemo → pCR improved; surgical feasibility maintained; higher R0 rate; safe perioperative profile.
KEYNOTE-671 (2023, N Engl J Med)	Randomized, double-blind, phase III trial (resectable stage II, IIIA, or IIIB [N2 node stage])	Pembrolizumab plus cisplatin vs. Placebo plus cisplatin chemotherapy alone → surgery → adjuvant pembrolizumab/placebo	797	642 completed surgery: 325 (IO+Chemo), 317 (Chemo)	566 radical (R0) resection: 299 (IO+Chemo), 267 (Chemo)	30-day mortality = 2/325 (0.6%) in IO+Chemo arm	Peri-op Pembro + Chemo → pCR improved; surgical feasibility preserved; R0 resection rate improved.
AEGEAN (2023, N Engl J Med)	Randomized, double-blind, phase III trial (resectable stage II to IIIB)	Durvalumab plus platinum vs. Placebo plus platinum chemotherapy alone → surgery → adjuvant durvalumab/placebo	802	571 completed surgery: 284 (IO+Chemo), 287 (Chemo)	531 radical (R0) resection: 269 (IO+ Chemo), 262 (Chemo)	Durvalumab arm: 9/400 (2.3%) death; Placebo arm: 2/402 (0.5%) death	Durva + Chemo (Peri-op) → pCR improved; surgical feasibility preserved; perioperative safety profile manageable.

NSCLC, non-small cell lung cancer; ITT, intention-to-treat; R0, microscopically margin-negative resection; IO, immunotherapy; ICI, immune checkpoint inhibitor; TKI, tyrosine kinase inhibitor; EGFR, epidermal growth factor receptor; ALK, anaplastic lymphoma kinase; CRT, chemoradiotherapy; RT, radiotherapy; SBRT, stereotactic body radiotherapy; OR, operating room; AE, adverse event; AEs, adverse events; GC, gemcitabine/cisplatin; NR, not reported.

**Table 6:** Pathological response and resection type across landmark neoadjuvant trials.

Study	Regimen type	N (ITT)	Resection	R0 Rate	MPR	pCR	Lobectomy	Bilobectomy	Pneumonectomy
NADIM (Provencio, 2020) (5)	IO + Chemo	46	41	41 (100%)	34 (82.9%)	26 (63.4%)	NR	NR	NR
NEOSTAR (Cascone, 2021) (16)	IO Mono	44	37	37 (100%)	13 (35.1%)	8 (21.6%)	NR	NR	NR
Shu et al. (2020) (18)	IO + Chemo	30	29	26 (89.7%)	17 (58.6%)	10 (34.5%)	19 (65.5%)	4 (13.8%)	3 (10.3%)
EORTC 08941 (Van Schil, 2005) (25)	Chemo	167	149	74 (49.7%)	NR	NR	58 (38.9%)		69 (46.3%)
Xiong et al. (2019) (28)	TKI Mono	19	14	13 (92.9%)	NR	NR	NR	NR	NR
EMERGING (Zhong, 2019) (17)	TKI vs Chemo	72	55	49 (89.1%)	3 (5.5%)	0 (0%)	43 (78.2%)	10 (18.2%)	2 (3.6%)
	TKI Mono	37	31	27 (87.1%)	3 (9.7%)	0 (0%)	24 (77.4%)	5 (16.1%)	2 (6.5%)
	Chemo	35	24	22 (91.7%)	0 (0%)	0 (0%)	19 (79.2%)	5 (20.8%)	0
RTOG-9309 (Albain, 2009) (3)	Chemo + CRT	202	164	144 (87.8%)	NR	NR	98 (59.8%)	NR	54 (32.9%)
NeoADAURA (Tsuboi, 2024/25)	TKI ± Chemo	358	331	307 (92.7%)	62 (18.7%)	15 (13.3%)	NR	NR	NR
	TKI+ Chemo	121	111	101 (90.1%)	31 (27.9%)	5 (4.5%)	NR	NR	NR
	TKI Mono	117	113	107 (94.7%)	29 (25.7%)	10 (8.8%)	NR	NR	NR
	Chemo	120	107	99 (92.5%)	2 (1.9%)	0 (0%)	NR	NR	NR
Durvalumab ± SBRT (Altorki, 2021)	IO ± SBRT	60	52	NR	18 (34.6%)	8 (15.4%)	38 (73.1%)	5 (9.6%)	9 (17.3%)
	IO Mono	30	26	NR	2 (7.7%)	0 (0%)	21 (80.8%)	1 (3.8%)	4 (15.4%)
	IO + SBRT	30	26	NR	16 (61.5%)	8 (30.8%)	17 (65.4%)	4 (15.4%)	5 (19.2%)
Pembro + CRT (Lemmon, 2022)	IO + CRT	9	6	6 (100%)	4 (66.7%)	4 (66.7%)	6 (100%)	0	0
ALNEO (Leonetti, 2024/25) (27)	TKI Mono	33	28	24 (85.7%)	15 (53.6%)	4 (14.3%)	21 (75.0%)	NR	3 (10.7%)
Camrelizumab + Chemo (Lei, 2023) (39)	IO ± Chemo	88	82	73 (89.0%)	35 (42.7%)	18 (22.0%)	NR	NR	12 (14.6%)
	IO + Chemo	43	40	37 (92.5%)	28 (70.0%)	14 (35.0%)	NR	NR	4 (10.0%)
	Chemo	45	42	36 (85.7%)	7 (16.7%)	4 (9.5%)	NR	NR	8 (19.0%)
CheckMate 816 (Forde, 2022) (4)	IO ± Chemo	358	284	229 (80.6%)	82 (28.9%)	47 (16.5%)	197 (69.4%)	7 (2.5%)	59 (20.8%)
	IO + Chemo	179	149	124 (83.2%)	66 (44.3%)	43 (28.9%)	115 (77.2%)	3 (2.0%)	25 (16.8%)
	Chemo	179	135	105 (77.8%)	16 (11.9%)	4 (3.0%)	82 (60.7%)	4 (3.0%)	34 (25.2%)
KEYNOTE-671 (Wakelee, 2023) (19)	IO ± Chemo	797	642	566 (88.2%)	164 (25.5%)	88 (13.7%)	494 (76.9%)	52 (8.1%)	76 (11.8%)
	IO + Chemo	397	325	299 (92.0%)	120 (36.9%)	72 (22.2%)	256 (78.8%)	26 (8.0%)	37 (11.4%)
	Chemo	400	317	267 (84.2%)	44 (13.9%)	16 (5.0%)	238 (75.1%)	26 (8.2%)	39 (12.3%)
AEGEAN (Heymach, 2023) (24)	IO ± Chemo	802	571	531 (93.0%)	168 (29.4%)	45 (7.9%)	459 (80.4%)	33 (5.8%)	56 (9.8%)
	IO + Chemo	400	284	269 (94.7%)	122 (43.0%)	35 (12.3%)	238 (83.9%)	13 (4.6%)	27 (9.5%)
	Chemo	402	287	262 (91.3%)	46 (16.0%)	10 (3.5%)	221 (77.0%)	20 (7.0%)	29 (10.1%)

Percentages of R0 rate, MPR, pCR, Lobectomy, Bilobectomy, Pneumonectomy are among resected. NSCLC, non-small cell lung cancer; ITT, intention-to-treat; R0, microscopically margin-negative resection; MPR, major pathological response; pCR, pathologic complete response; IO, immunotherapy; TKI, tyrosine kinase inhibitor; EGFR, epidermal growth factor receptor; ALK, anaplastic lymphoma kinase; CRT, chemoradiotherapy; SBRT, stereotactic body radiotherapy; CT, chemotherapy; Osi, osimertinib; Pbo, placebo; NR, not reported.

#### 3.9.1 Surgical resection and R0 rates

Across these cohorts, the surgical endpoint data suggest a marked improvement in R0 resection rates over time. In the pre-immunotherapy era, exemplified by EORTC 08941, R0 resection was achieved in 49.7% of patients who proceeded to surgery (25). Among patients who proceeded to resection, NADIM (5) and NEOSTAR (16) reported R0 resection in all resected cases (100%). Even in larger randomized phase III, targeted based therapy settings such as NeoADAURA (26), the R0 rate reached 92.7%. Differences in R0 resection rates were also observed among regimens. Generally, the R0 resection rate is higher in the immunotherapy plus chemotherapy group than in the chemotherapy-alone group. This consistent advantage across multiple phase III trials [CheckMate 816 (4), KEYNOTE-671 (6), AEGEAN (24)] suggests that immunotherapy-containing regimens not only deepen pathological response but also enhance the likelihood of achieving complete tumor clearance at the time of surgery.

#### 3.9.2 Surgical approaches and operative patterns

A clinically relevant signal for operative planning is the apparent shift in extent of resection. Earlier neoadjuvant trials were characterized by substantial pneumonectomy use, reaching approximately 46.3% in EORTC 08941 (25) and 32.9% in RTOG 9309 (3). In the more contemporary cohorts summarized here, pneumonectomy was less frequently reported., accompanied by a high proportion of lobectomy. For example, pneumonectomy rate was 10.3% and lobectomy rate was 65.5% in Shu et al. (18). These data are consistent with an evolving pattern toward lung-sparing resections in the modern neoadjuvant era. Moreover, pneumonectomies were less common in the immunotherapy-plus-chemotherapy group than in the chemotherapy-alone group. This trend toward parenchymal preservation carries important implications: it may translate into reduced perioperative risk and better long-term functional outcomes for patients.

#### 3.9.3 Efficacy stratification

Across regimens, the data also highlight differences in pathological response profiles. Targeted therapy cohorts [NeoADAURA (26) and ALNEO (27)], specifically the TKI monotherapy arms, achieved high R0 rates (94.7% and 85.7%) but reported relatively low pCR rates (8.8% and 14.3%) across arms. By contrast, chemo-immunotherapy cohorts [NADIM (5) and Shu et al. (18)] reported substantially higher pCR rates (63.4% and 34.5%). From a surgical perspective, this contrast underscores that neoadjuvant TKI strategies may still leave residual viable tumor requiring meticulous anatomic resection, whereas immunotherapy-based combinations more frequently achieve deeper pathological responses.

A similar gradient was observed when comparing immunotherapy monotherapy with immunotherapy plus chemotherapy. In immunotherapy monotherapy cohorts [NEOSTAR (16)], pCR reached to 21.6%. In cohorts receiving immunotherapy plus chemotherapy, pCR reached to 63.4% [NADIM (5)] While monotherapy can induce immune-mediated regression, these data suggest that the addition of chemotherapy is associated with higher pCR rates in resected specimens across the summarized studies (**Tables 5**, **6**).

### 3.10 Topic modeling analysis (LDA validation of thematic structure)

To evaluate the semantic coherence of the bibliometric clustering results, LDA was applied to the abstracts and keywords of all included publications. The model yielded eight latent topics (T0–T7), each corresponding to a distinct thematic domain within neoadjuvant therapy research in lung cancer.

#### 3.10.1 Topic distribution and semantic composition

**Figure 9:**
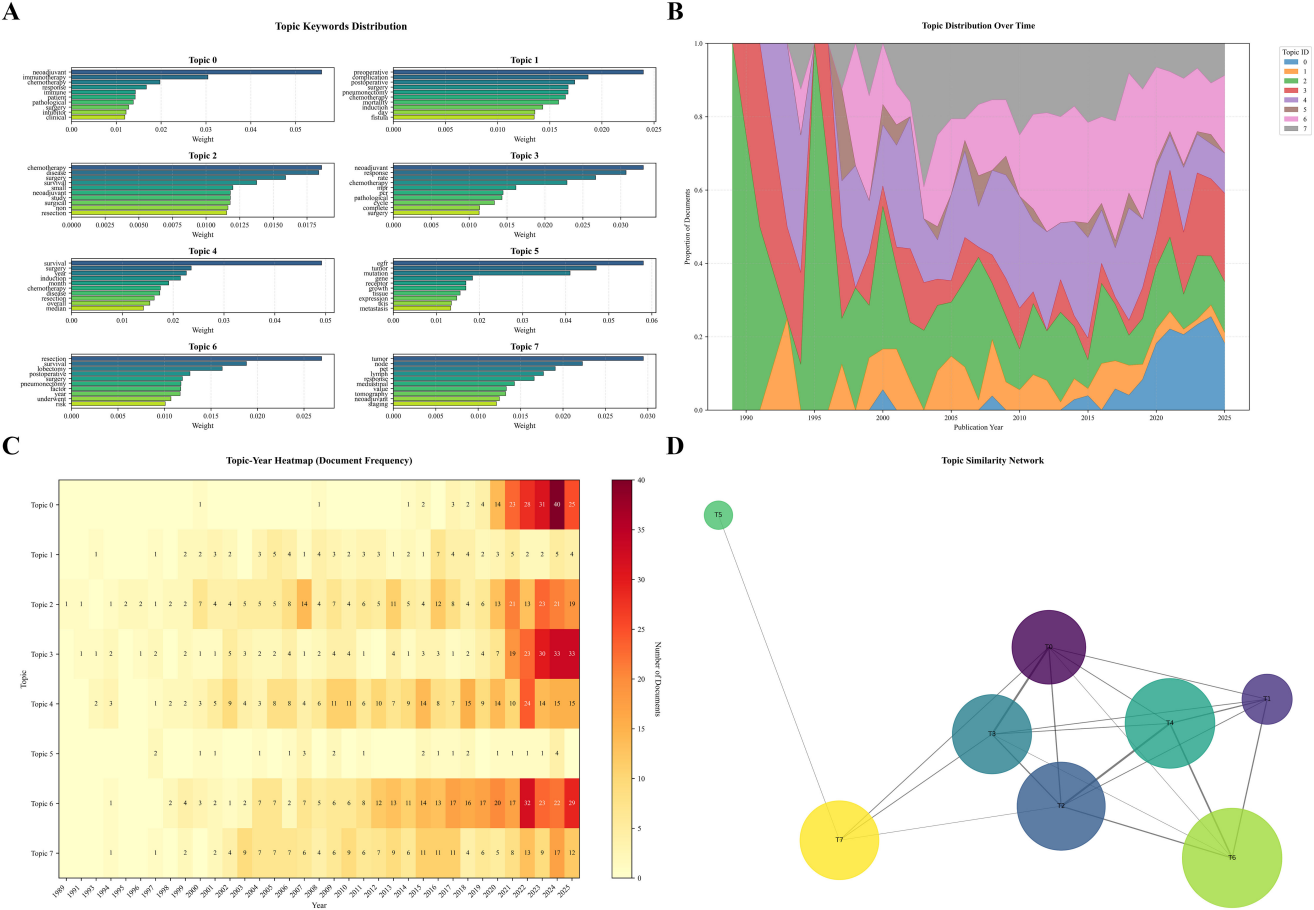
Temporal and semantic evolution of research topics in neoadjuvant therapy for lung cancer (1989–2025) by LDA methods. **(A)** Representative topic keyword distributions for T0-T7, showing the 10 highest-weighted terms per topic. **(B)** Topic-time area chart based on LDA modeling, showing proportional evolution of eight topics (T0-T7) from 1989 to 2025. **(C)** Topic-year heatmap illustrating document frequency by topic across years (T0-T7). **(D)** Topic similarity network visualizing semantic proximity among the eight topics.

As shown in **Figure 9A** and **Table 7**, Topic 0 (T0; “neoadjuvant immunotherapy, chemotherapy, pathological response, surgery”) accounted for the largest share in the most recent decade, consistent with the expansion of perioperative immune checkpoint blockade trials. Topic 1 (T1) captured perioperative complications and surgical feasibility (preoperative, complication, postoperative, pneumonectomy), reflecting growing attention to safety and operability. Topic 2 (T2) was dominated by conventional chemotherapy and survival-related terms (chemotherapy, disease, surgery, survival), whereas Topic 3 (T3) concentrated on pathological efficacy endpoints (pCR, MPR, response rate). Topic 4 (T4) emphasized long-term outcomes and survival analysis. Topic 5 (T5) reflected molecularly targeted therapy (EGFR, mutation, TKI). Topic 6 (T6) focused on operative techniques and postoperative outcomes. Topic 7 (T7) represented imaging and staging integration (PET, tomography, mediastinal node evaluation). Overall, the semantic content of these topics closely paralleled the thematic clusters derived from CiteSpace, supporting the internal consistency of the knowledge structure from both bibliometric and text-based perspectives.

**Table 7:** Topic summary in latent Dirichlet allocation topic modeling.

Topic ID	Top keywords	Document count	Mean year
Topic 0	neoadjuvant, immunotherapy, chemotherapy, response, immune, patient, pathological, surgery, inhibitor, clinical	175	2022.2
Topic 1	preoperative, complication, postoperative, surgery, pneumonectomy, chemotherapy, mortality, induction, day, fistula	81	2012.7
Topic 2	chemotherapy, disease, surgery, survival, small, neoadjuvant, study, surgical, non, resection	250	2014.8
Topic 3	neoadjuvant, response, rate, chemotherapy, mpr, pcr, pathological, cycle, complete, surgery	202	2018.7
Topic 4	survival, surgery, year, induction, month, chemotherapy, disease, resection, overall, median	259	2014.5
Topic 5	egfr, tumor, mutation, gene, receptor, growth, tissue, expression, tkis, metastasis	26	2012.9
Topic 6	resection, survival, lobectomy, postoperative, surgery, pneumonectomy, factor, year, underwent, risk	319	2016.9
Topic 7	tumor, node, pet, lymph, response, mediastinal, value, tomography, neoadjuvant, staging	200	2014.4

#### 3.10.2 Temporal evolution and thematic transition

The area chart (**Figure 9B**) and topic-year heatmap (**Figure 9C**) indicate a clear thematic transition after 2018. Prior to 2015, topics related to chemotherapy, surgical technique, and long-term survival (T2, T4, and T6) were predominant. From 2018 onward, immunotherapy-associated themes (T0 and T3) expanded rapidly, accounting for nearly half of all publications by 2023. This temporal shift aligns with the emergence of pivotal neoadjuvant immunotherapy or immunochemotherapy trials (e.g., NADIM, NEOSTAR, and CheckMate 816), highlighting a broader transition from cytotoxic induction strategies toward immune-based perioperative approaches and pathology-driven endpoints.

#### 3.10.3 Topic interactions and interdisciplinary convergence

The topic similarity network (**Figure 9D**) demonstrates dense interconnections among T0 (immunotherapy), T2 (chemotherapy), T3 (pCR/MPR), and T4 (survival), forming a central hub that links therapeutic strategies with surgical and pathological endpoints. In contrast, T5 (targeted therapy) and T7 (imaging-staging) were positioned more peripherally, suggesting greater thematic specialization and comparatively slower integration into the dominant perioperative paradigm. Together, these interaction patterns are consistent with cross-disciplinary convergence across oncology, thoracic surgery, and immunology within the contemporary neoadjuvant literature.

## 4 Discussion

This comprehensive bibliometric and scientometric analysis provides an integrated overview of global research trends in neoadjuvant therapy and surgery for lung cancer over the past decades. By leveraging multiple analytical platforms, including bibliometrix, VOSviewer, CiteSpace, and LDA topic modeling, the study delineates the evolution of publication output, intellectual structure, collaboration networks, and thematic transitions within this rapidly developing domain. Collectively, the findings suggest that research on neoadjuvant therapy for lung cancer has shifted from conventional chemotherapy-based induction toward a biomarker- and immunotherapy-driven perioperative paradigm, reflecting both scientific maturation and closer translational alignment between surgical and systemic oncology.

### 4.1 Global research evolution and publication dynamics

The substantial growth of publications, particularly after 2018, aligns temporally with the clinical introduction and validation of immune checkpoint inhibitors (ICIs) in the perioperative setting. A compound annual growth rate of approximately 14–15% underscores an evolving research landscape, and the citation trajectory further indicates a transition from classic resectability and survival trials toward immuno-oncology-oriented perioperative management. This inflection reflects the convergence of two historically distinct research tracks, thoracic surgery and systemic immunotherapy, into a more unified translational continuum in curative-intent treatment.

### 4.2 Geographic collaboration

At the national level, China and the United States emerged as the leading contributors, together producing over half of the total global output. China’s publication surge is consistent with rapid clinical adoption and intensive investigation of neoadjuvant immunotherapy; however, its lower MCP ratio suggests potential room to expand international collaboration. The United States remains prominent in total citations, network centrality, and cross-regional linkages. European countries such as Germany, Italy, and Spain also show substantial normalized coupling, which may be facilitated by European Union supported research frameworks.

### 4.3 Evolution of research focus and conceptual transformation

Early neoadjuvant studies emphasized cytotoxic chemotherapy and chemoradiotherapy induction regimens such as EORTC 08941 and INT-0139, with primary aims centered on resectability and survival. Keyword burst analysis highlighted terms including “cisplatin”, “radiotherapy”, and “survival”, consistent with a conventional framework that prioritized long-term outcomes over pathological response metrics. With the emergence of ICIs in the neoadjuvant setting, the thematic architecture shifted. The largest co-citation cluster (#0: neoadjuvant chemoimmunotherapy) and the dominant LDA topic both emphasized “immunotherapy”, “pathological response”, and “surgery”. This pattern corresponds to pivotal phase II–III trials (e.g., NADIM, CheckMate 816, and NEOSTAR) that brought pCR and MPR into the center of perioperative research. Accordingly, the field has increasingly adopted biologically oriented endpoints, reflecting a conceptual transition from anatomical downstaging toward deeper tumor clearance before resection, while continuing to evaluate how these endpoints relate to longer-term outcomes.

### 4.4 Integration of immunotherapy and surgical feasibility

Surgical endpoint mapping indicates that contemporary immunotherapy-based induction cohorts frequently report high surgical completion rates and high R0 resection rates, with perioperative feasibility generally maintained in the reported series. In contrast to historical chemoradiation protocols, where pneumonectomy and perioperative risk were recurring concerns, modern ICI-based regimens are commonly described as operable within multidisciplinary pathways, with safety profiles often comparable to chemotherapy controls in the available trial reports.

In parallel, profound downstaging and improved operability have coincided with a trend toward lung-sparing resections. Pneumonectomy-related signals have decreased from nearly 50% in the chemotherapy era to below 10% in more contemporary cohorts. This shift is clinically meaningful because it supports a paradigm in which lobectomy is increasingly feasible for selected patients who might previously have required total lung removal. Functional preservation may translate into reduced perioperative risk and long-term respiratory morbidity, particularly relevant to stage III resections, although such clinical advantages should be interpreted in light of cross-trial heterogeneity and differences in surgical candidacy.

It is important to emphasize that these trial-level comparisons are descriptive and hypothesis-generating. Direct comparisons across studies are confounded by differences in patient selection, trial design, staging criteria, surgical standards, and time period. The observed trend toward lower pneumonectomy rates in immunotherapy-based induction cohorts should be interpreted as a hypothesis-generating signal that warrants prospective evaluation in matched or randomized settings, rather than as direct evidence of regimen superiority.

### 4.5 Operative implications beyond response metrics

Taken together, these bibliometric and trial-derived signals support several surgery-facing inferences that should be interpreted as hypothesis-generating rather than meta-analytic estimates. First, increasing pCR/MPR reporting may be associated with a larger proportion of patients proceeding to definitive resection. However, it does not obviate core oncologic principles, including anatomic resection with systematic nodal dissection, because radiologic response may not fully reflect residual viable tumor. Second, perioperative decision-making should explicitly distinguish radiographic response from resectability. Persistent mass-like findings after induction immunotherapy may reflect fibrosis, necrosis, or immune-cell infiltration; therefore, resectability assessment should be based on multidisciplinary review and, when indicated, metabolic imaging and invasive mediastinal staging rather than radiologic appearance alone.

Based on these signals, several practical considerations may be helpful for thoracic surgeons managing stage II–III candidates in contemporary perioperative pathways: Patient selection: prioritize multidisciplinary assessment, integrating radiologic staging, invasive mediastinal evaluation, actionable molecular drivers (e.g., EGFR), and the anticipated likelihood of benefit from immunotherapy-containing regimens (28, 29). Extent of resection: consider lung-sparing strategies when downstaging permits, while maintaining oncologic principles (complete resection and systematic nodal dissection) despite high pCR/MPR signals (30, 31). Surgical approach: minimally invasive resection may be feasible in selected patients. However, post-immunotherapy fibrosis and adhesions can increase technical complexity. Surgeons should plan for secure vascular control and maintain a low threshold for conversion when safety is threatened (31, 32). Imaging interpretation: residual radiologic findings after neoadjuvant immunotherapy should not be equated with unresectability. Instead, imaging should be interpreted in conjunction with metabolic assessment, nodal evaluation, and the overall clinical trajectory (33, 34). Biomarkers: ctDNA/minimal residual disease (MRD) is an emerging adjunct for recurrence risk estimation and trial stratification (35). Its role in routine operative decision-making remains investigational pending prospective validation and assay standardization.

### 4.6 Emergence of molecular and translational dimensions

Recent keyword bursts in “ctDNA”, “TMB”, and “immune-related adverse events” signal an expanding molecular and translational layer in perioperative research. Cluster patterns highlight dynamic ctDNA monitoring as a promising approach to infer MRD and to study postoperative recurrence risk. Recent translational studies have provided empirical support for these bibliometric signals. Zhai et al. demonstrated that a plasma exosomal miRNA signature can effectively predict pathological complete response in NSCLC patients receiving neoadjuvant immunochemotherapy, with the fatty acid biosynthesis pathway emerging as a key metabolic nexus linking tumor metabolism and immune function (36). Beyond static biomarker assessment, the CLEVER study—a prospective multi-region sampling cohort of Chinese NSCLC patients—integrates multi-omics profiling and personalized MRD tracking to map clonal evolution and microenvironment remodeling under neoadjuvant therapy (37). This paradigm of dynamic molecular monitoring, linking clonal dynamics to clinical outcomes, is precisely the transition captured by our keyword burst analysis, which identified “ctDNA” and “MRD” as rapidly intensifying research fronts.

The convergence of molecular diagnostics, immunology, and surgical oncology in both co-citation and topic networks reflects cross-disciplinary maturation and supports ongoing efforts toward biomarker-informed decisions regarding patient selection, timing, and intensity of perioperative therapy, areas that will require standardized assays and prospective validation for routine implementation.

### 4.7 Methodological and interdisciplinary perspectives

The combined use of CiteSpace for structural network mapping and LDA for semantic modeling represents a methodologically complementary framework in bibliometric research. This dual approach enables simultaneous characterization of intellectual linkages and thematic semantics, thereby strengthening interpretability. The observed correspondence between highly modular co-citation clusters and LDA-derived topics, particularly around “neoadjuvant immunotherapy” and “pathologic response”, supports the coherence of the field’s intellectual evolution. Interdisciplinary coupling among oncology, thoracic surgery, and immunology appears to have accelerated translational progress and contributed to a globally connected research ecosystem.

### 4.8 Strengths, limitations, and future directions

A key strength of this study is the integration of multiple bibliometric frameworks, enabling cross-validation between quantitative indicators (NP, TC, h-index) and relational metrics (total link strength, modularity, and coupling). Several limitations should be acknowledged. First, although we selected three major databases (WoS, PubMed, and Wanfang) to capture both English and Chinese literature, the exclusion of other important databases such as Scopus and Embase may introduce a risk of language and regional publication bias. Second, author disambiguation and citation lag can affect impact estimation, particularly for recently published trials and emerging themes. Third, bibliometric prominence primarily reflects patterns of academic attention and investment; high citation counts or keyword bursts should not be interpreted as evidence of clinical superiority of one regimen over another. Fourth, we did not perform a formal quality assessment or risk-of-bias evaluation on the included publications. All studies, regardless of their design, were assigned equal weights, which may affect the reliability of the results. Finally, while LDA captures semantic regularities at scale, it does not resolve contextual nuances such as clinical heterogeneity, differences in trial design, or regimen-specific eligibility criteria; therefore, topic-derived patterns should be interpreted as descriptive signals rather than comparative effectiveness estimates.

## 5 Conclusions

In summary, neoadjuvant therapy research for resectable lung cancer has undergone a transformation from cytotoxic induction toward precision immuno-oncology integrated with surgery. By linking citation networks with semantic-level topic evolution, this study provides an analytical framework to monitor future developments in perioperative cancer research and highlights the ongoing transition of thoracic oncology toward a stage defined by precision, collaboration, and translational continuity.

## Data Availability

Publicly available datasets were analyzed in this study. This data can be found here: The data used and analyzed in this study are available from the corresponding author on reasonable request.

## Supplementary material

Supplementary Table 1 TITAN-Guideline-Checklist-2025

Supplementary Table 2 Search Strategy

Supplementary Table 3 Operating parameters for CiteSpace and VOSviewer analyses

Supplementary Table 4 Synonym thesaurus for keyword standardization

Supplementary Figure 1 K-perplexity curve for topic number selection

Supplementary Figure 2 Bradford’s law partition (Zones 1–3) based on cumulative output (cumFreq), showing a compact core and a long tail.

Supplementary Figure 3 Distribution of corresponding authors by number of publications and local citation counts

Supplementary Figure 4 Sensitivity analysis of keyword co-occurrence thresholds for network visualization.

Supplementary Figure 5 Model selection criteria for the Bai-Perron structural break test applied to the annual publication series (1989–2025).
