# Supplementary material for: Global research trends and thematic evolution in neoadjuvant therapy and surgery for non-small cell lung cancer: a bibliometric and scientometric analysis
Source: Front Immunol. 2026 Jul 29;17:1900455. doi: 10.3389/fimmu.2026.1900455 (PMC13461830; doi:10.3389/fimmu.2026.1900455)
Supplement: Supplementary file 2 [file Table2.docx]

**Supplemental Table 2 Search strategy**

| **Database** | **Search query (database-specific syntax)** | **Time span / date limits** | **Additional filters / notes** |
| --- | --- | --- | --- |
| Web of Science Core Collection | TS=("lung cancer" OR "pulmonary neoplasm*" OR "bronchogenic carcinoma" OR "lung neoplasm*") AND TS=("neoadjuvant therapy" OR "neoadjuvant treatment" OR "preoperative therapy" OR "preoperative treatment" OR "induction therapy" OR "induction treatment") AND TS=("surgery" OR "resection" OR "operation" OR "surgical resection" OR "surgical procedure*") | Timespan: 1989–2025 | Search field: Topic (TS). |
| PubMed | (("Lung Neoplasms"[MeSH Terms] OR "Lung Cancer"[Title/Abstract] OR "Pulmonary Neoplasms"[Title/Abstract] OR "Bronchogenic Carcinoma"[Title/Abstract] OR "Lung Tumor"[Title/Abstract])AND ("Neoadjuvant Therapy"[MeSH Terms] OR "Preoperative Therapy"[Title/Abstract] OR "Induction Therapy"[Title/Abstract] OR "Neoadjuvant Treatment"[Title/Abstract] OR "Preoperative Treatment"[Title/Abstract])AND ("Surgical Procedures, Operative"[MeSH Terms] OR "Surgery"[Title/Abstract] OR "Surgical Resection"[Title/Abstract] OR "Operation"[Title/Abstract] OR "Surgical Intervention"[Title/Abstract]))AND ("1989/01/01"[PDAT] : "2025/12/31"[PDAT]) AND "English"[Lang] | ("1989/01/01"[PDAT] : "2025/12/31"[PDAT]) | Language: English ("English"[Lang]). |
| Wanfang | (((("lung cancer" OR "lung tumor" OR "bronchial carcinoma" OR "bronchogenic carcinoma") AND ("neoadjuvant therapy" OR "preoperative therapy" OR "induction therapy" OR "preoperative adjuvant therapy")) AND ("surgery" OR "surgical resection" OR "surgical operation" OR "surgical procedure")) | 1989 TO 2025 | Language: Chinese |
