# Supplementary material for: Global research trends and thematic evolution in neoadjuvant therapy and surgery for non-small cell lung cancer: a bibliometric and scientometric analysis
Source: Front Immunol. 2026 Jul 29;17:1900455. doi: 10.3389/fimmu.2026.1900455 (PMC13461830; doi:10.3389/fimmu.2026.1900455)
Supplement: Supplementary file 3 [file Table3.docx]

**Supplementary Table 3**. Operating parameters for CiteSpace and VOSviewer analyses

| Parameter category | CiteSpace (version 6.4.R2) | VOSviewer (version 1.6.20) |
| --- | --- | --- |
| Time slicing | 1989–2025, 1-year per slice | Not applicable |
| Selection thresholds | Top N = 50 per slice (for cited references); Top N = 30 per slice (for keywords) | Keyword: minimum occurrences = 10; Author/country: minimum documents = 5; Source: minimum documents = 3 |
| Pruning method | Pathfinder network scaling + minimum spanning tree | Not applicable; default association strength normalization |
| Clustering algorithm | Log-likelihood ratio (LLR) for cluster labeling; K-means clustering (K = 25) for network grouping | Linlog/modularity-based clustering (resolution parameter = 1.0, minimum cluster size = 3) |
| Modularity Q and Silhouette S | Q = 0.712; S = 0.936 (for co-citation network) | Not applicable |
| Citation burst detection | Kleinberg's burst detection algorithm; γ = 1.0, e = 2.0 | Not applicable |
| Label extraction | Noun phrases from titles and abstracts | Not applicable |
| Visualization layout | Default layout algorithm | Attraction = 3, Repulsion = 0 |
| Normalization method | Cosine similarity | Association strength |
