# Supplementary material for: Global research trends and thematic evolution in neoadjuvant therapy and surgery for non-small cell lung cancer: a bibliometric and scientometric analysis
Source: Front Immunol. 2026 Jul 29;17:1900455. doi: 10.3389/fimmu.2026.1900455 (PMC13461830; doi:10.3389/fimmu.2026.1900455)
Supplement: Supplementary file 4 [file Table4.docx]

Supplementary Table 4. Synonym thesaurus for keyword standardization

| Standardized term | Synonyms merged |
| --- | --- |
| PD-L1 | PD-L1, PDL1, Programmed Death-Ligand 1, CD274, B7-H1 |
| PD-1 | PD-1, PDL1, Programmed Death-1, CD279 |
| CTLA-4 | CTLA-4, CTLA4, CD152 |
| EGFR | EGFR, Epidermal Growth Factor Receptor, HER1, ErbB1 |
| TKI | TKI, Tyrosine Kinase Inhibitor, TKIs |
| ctDNA | ctDNA, circulating tumor DNA, circulating tumour DNA |
| MRD | MRD, minimal residual disease, molecular residual disease |
| pCR | pCR, pathologic complete response, pathological complete response |
| MPR | MPR, major pathologic response, major pathological response |
| NSCLC | NSCLC, non-small cell lung cancer, non-small cell lung carcinoma |
| SCLC | SCLC, small cell lung cancer, small cell lung carcinoma |
| ICI | ICI, immune checkpoint inhibitor, immune checkpoint inhibitors, checkpoint inhibitor |
